# Mass Spectrometry-Based Proteomic and Immunoproteomic Analyses of the *Candida albicans* Hyphal Secretome Reveal Diagnostic Biomarker Candidates for Invasive Candidiasis

**DOI:** 10.3390/jof7070501

**Published:** 2021-06-23

**Authors:** Catarina Vaz, Aida Pitarch, Emilia Gómez-Molero, Ahinara Amador-García, Michael Weig, Oliver Bader, Lucía Monteoliva, Concha Gil

**Affiliations:** 1 Department of Microbiology and Parasitology, Faculty of Pharmacy, Complutense University of Madrid and IRYCIS, 28040 Madrid, Spain; cataoliv@ucm.es (C.V.); apitavel@ucm.es (A.P.); ahinaram@ucm.es (A.A.-G.); conchagil@ucm.es (C.G.); 2 Institute for Medical Microbiology and Virology, University Medical Center Gottingen, Kreuzbergring 57, D-37075 Gottingen, Germany; emiliagomez803@hotmail.com (E.G.-M.); mweig@gwdg.de (M.W.); oliver.bader@med.uni-goettingen.de (O.B.); 3 Proteomics Facility, Faculty of Pharmacy, Complutense University of Madrid, 28040 Madrid, Spain

**Keywords:** *Candida albicans*, invasive candidiasis, secretome, secreted proteins, hypha, diagnosis, biomarkers, immunoproteomics, SERPA, serologic response

## Abstract

Invasive candidiasis (IC) is associated with high morbidity and mortality in hospitalized patients if not diagnosed early. Long-term use of central venous catheters is a predisposing factor for IC. Hyphal forms of *Candida albicans* (the major etiological agent of IC) are related to invasion of host tissues. The secreted proteins of hyphae are involved in virulence, host interaction, immune response, and immune evasion. To identify IC diagnostic biomarker candidates, we characterized the *C. albicans* hyphal secretome by gel-free proteomic analysis, and further assessed the antibody-reactivity patterns to this subproteome in serum pools from 12 patients with non-catheter-associated IC (ncIC), 11 patients with catheter-associated IC (cIC), and 11 non-IC patients. We identified 301 secreted hyphal proteins stratified to stem from the extracellular region, cell wall, cell surface, or intracellular compartments. ncIC and cIC patients had higher antibody levels to the hyphal secretome than non-IC patients. Seven secreted hyphal proteins were identified to be immunogenic (Bgl2, Eno1, Pgk1, Glx3, Sap5, Pra1 and Tdh3). Antibody-reactivity patterns to Bgl2, Eno1, Pgk1 and Glx3 discriminated IC patients from non-IC patients, while those to Sap5, Pra1 and Tdh3 differentiated between cIC and non-IC patients. These proteins may be useful for development of future IC diagnostic tests.

## 1. Introduction

*Candida albicans* is an opportunistic fungal pathogen that can grow as yeast-form, pseudohyphal, or hyphal cells, each under specific host or environmental stimuli. Hyphal forms are the most often observed morphology of this polymorphic fungus in tissue biopsies and histological analysis of clinical lesions [1]. It appears to be linked to the hyphae increased ability to adhere to host cells and invade host tissues than do other morphotypes [2,3]. In contrast, yeast-form cells usually contribute to bloodstream dissemination [4].

*C. albicans* is the major etiologic agent of invasive candidiasis (IC), which is associated with high morbidity and mortality in immunocompromised and critically ill patients [5,6]. There are several predisposing factors for IC. These are related to (i) host microbiota alteration (e.g., long-term treatment with broad-spectrum antibiotics), (ii) rupture of the cutaneous and gastrointestinal barriers (e.g., organ transplants or use of central venous catheters), and (iii) impairment of host defenses (e.g., cancer, neutropenia, chemotherapy or corticosteroid therapy) [5,7]. Colonization is a prerequisite for acquisition of IC [8]. Long-term use of central venous catheters enables *Candida* cells to translocate from mucocutaneous sites into the bloodstream, facilitating invasion of host tissues and organs. Catheter-related bloodstream infections caused by *Candida* spp. are common and show life-threatening complications [9,10,11].

Detection of IC at an early stage of the disease is crucial for optimizing antifungal therapy and improving the clinical outcomes of patients. The gold standards for diagnosing IC are still blood culture with subsequent species identification and tissue biopsies. However, blood cultures can take 2–5 days to attain a decisive result and are not useful for the diagnosis of deep-seated candidiasis in the absence of candidemia; in contrast histopathological examination is often a too invasive method for critically ill patients [7,12]. Matrix-assisted laser desorption-time of flight mass spectrometry (MALDI-TOF MS) is a useful technology for the identification of *C. albicans* species, but requires positive cultures [13]. PCR-based technologies used for *Candida* DNA amplification have high specificity and sensitivity for IC diagnosis, but lack standardized protocols [14]. The T2 magnetic resonance (T2Candida) assay is a new diagnostic method of IC that accurately detects the most common pathogenic *Candida* species [15,16], but its sensitivity decreases in the absence of intact *Candida* cells in whole-blood samples [17]. Other non-culture methods mainly include the detection of *Candida* mannans and 1,3-β-D-glucan as well as host-derived biomarkers, such as human antibodies against *Candida* antigens [7]. There is therefore a general effort being made for the discovery of novel useful biomarkers, including the detection of suitable biomolecules both from pathogen and from the host [7]. These could be used as a faster diagnostic method because there is no need to culture the microorganism.

Classical immunoproteomics or serological proteome analysis (SERPA) are widely used techniques for screening panels of antibody biomarkers for diverse infectious diseases [18,19,20,21,22,23]. It enables the characterization of the immunome of any microorganism, which is the subset of its proteome targeted by the immune system [24]. Our group has applied this technique, which combines two-dimensional gel electrophoresis (2-DE) with Western blotting and mass spectrometry (MS), to identify biomarker candidates of IC [24,25,26], and has previously shown that the use of multiple biomarker panels should be the path to choose for development of better diagnostic and prognostic assays for IC [27,28,29]. Most of immunoproteomic studies for the search of new biomarker candidates for IC were performed with intracellular or cell wall proteins from *C. albicans* [24,25,30,31,32]. A SERPA study used the secretome of *C. albicans* yeasts and hyphae as a source of antigens, and showed that hyphal secreted proteins were more abundant and immunogenic than yeast secreted proteins [33]. Despite the reduced number of sera used, this study identified a core set of antibody-mediated reactivity against 19 *C. albicans* secreted proteins, seven of which could serve for the diagnosis of candidemia (Xog1, Lip4, Asc1, Met6, Tsa1, Tpi1, and Prx1).

*C. albicans* secretes many proteins involved in the degradation of host proteins, lipids, or carbohydrates, acquisition of zinc or other essential ions, and protecting against microbial peptides, among others [34,35,36,37]. Proteins can be secreted either by the classical secretory pathway or by alternative routes of exportation [38]. Our group has described the secretion of classical cytoplasmic proteins (lacking a signal peptide) inside extracellular vesicles (EV) [35,39]. Some of these proteins are ‘moonlighting’ as they have different functions depending on their subcellular location [40,41]. Fungal EVs also interact with the host [42]. A recent work has demonstrated that the biofilms of *C. albicans* defective mutants in the endosomal sorting complexes produced less EVs, and these biofilms were more sensitive to antifungal drugs, which helped to understand the role in EVs in cell-cell signaling [43]. Taking into account the important role of secreted proteins during interaction with the host, these could be useful for the future design of vaccine strategies and diagnostic tests for IC [35,39].

In this work, we performed a proteomic analysis to characterize the *C. albicans* hyphal secretome. We also examined the serological response to this subproteome in patients with IC associated or not with catheters with the purpose of identifying potential biomarker candidates for IC diagnosis.

## 2. Materials and Methods

### 2.1. Study Population and Serum Samples

Serum specimens were obtained from the Institute for Medical Microbiology and Virology, University Medical Center Göttingen in Germany. These were pooled into three groups: (i) the non-catheter-associated IC (ncIC) group, which included sera from 12 ncIC patients, (ii) the catheter-associated IC (cIC) group, which comprised sera from 11 cIC patients, and (iii) the negative control (non-IC) group, which encompassed sera from 11 matched patients with similar age and sex and being from the same hospital wards as those from ncIC or cIC, but without IC. ncIC was defined as isolation of the same *Candida* species in one or more blood cultures and/or in culture from a sterile liquid or organ. Patients were defined as having cIC if they had the same *Candida* species in one or more catheter cultures. Only sera from patients that had previously been tested negative for HIV and hepatitis were included. The use of these sera in diagnostic research areas was approved by the University Ethics Committee (UMG-17/9/08) and patients consented into the use of left-over diagnostic material. All sera were stored at −80 °C until use. Baseline characteristics of patients are shown in Table S1.

### 2.2. Isolation of C. albicans Hyphal Secreted Proteins

*C. albicans* strain SC5314 was maintained in yeast extract-peptone-dextrose (YPD) plates (2% glucose, 1% yeast extract, 2% peptone, and 2% agar) at 30 °C. Cells from a yeast colony were grown overnight in synthetic defined (SD) medium supplemented with amino acids (2% glucose, 0.5% ammonium sulfate, 0.17% yeast nitrogen base, 0.19% amino acids mix without uracil, and 0.01% uracil) at 180 rpm and 30 °C. Pre-inoculum was adjusted to an optical density of 0.1, and incubated for 6 h. Cells were recovered, washed in PBS, and counted in Neubauer chamber. Then, 5 × 10^5^ cells/mL were incubated for 18 h at 100 rpm and 37 °C in two different media: (i) salt medium+GlcNac, which included salt medium (0.45% NaCl, 0.08% yeast nitrogen base, 0.25% ammonium sulfate, pH 7.4) supplemented with 2.5 mM N-acetylglucosamine (GlcNac) [44]; and (ii) Lee medium, pH 6.7 [45]. Cells were centrifuged at 5000 rpm (without break) for 40 min at 4 °C, and the supernatant was recovered. From this moment forward, all steps were performed on ice. Hyphal morphology was confirmed by contrast microscopy, and cell lysis by propidium iodide (PI) staining. The supernatant was double filtered using 0.45 µm low protein binding filters (Millipore, Bedford, MA, USA) to eliminate cells that were not yet removed by centrifugation. One tablet of protease inhibitor cocktail (Thermo Fisher Scientific, Waltham, MA) dissolved in water and 0.1 M phenylmethanesulfonyl fluoride (PMSF) was added to the filtrate. One mL of the filtrate was plated and when one or more colony forming unit (CFU) grew on YPD plates, the supernatant was discarded. A two-step concentration was performed to increase the protein yield obtained and to remove contaminants (including salts and detergents). Supernatant containing the secreted proteins was concentrated approximately 100 times using centrifugal filter devices (10 kDa-pore Centricon Plus-70, Millipore, Burlington, MA, USA). The supernatant was frozen until precipitation. This was performed by incubation of the sample with a fourth part of trichloroacetic acid (TCA; 100% *w/v*) for 1 h in ice. Then, it was centrifuged at 10,000× *g* for 30 min at 4 °C. Supernatant was discarded. Ultrapure water was added to the pellet and vortexed. After this, chilled washing buffer from the 2-D clean-up kit (GE Healthcare, Buckinghamshire, UK) was added with 5 µL of wash additive, vortexed and kept at −20 °C overnight. Protein sample was then centrifuged, and the supernatant discarded. The pellet was dried (no longer than 5 min) and resuspended in rehydration buffer (7 M urea, 2 M thiourea, 2% 3-[(3-cholamidopropyl)dimethyl-ammonio]-1-propane sulfonate (CHAPS), 65 mM dithioerythritol (DTE), 0.5% immobilized pH gradient (IPG) buffer pH 3–10 (GE Healthcare), and 0.002% bromophenol blue) [46]. The different obtained secretomes were pooled in two batches (S1 and S2).

### 2.3. Protein Separation by Sodium Dodecyl Sulfate-Polyacrylamide gel Electrophoresis (SDS-PAGE)

The different secretome samples were separated by SDS-PAGE following standard protocols. Gels were stained either with a silver stain kit (BioRad, Hercules, CA, USA) or with Coomassie brilliant blue as follows. Briefly, gels were fixed with 50% methanol and 10% glacial acetic acid. Then, gels were stained with 0.1% Coomassie brilliant blue R-250, 50% methanol and 10% acetic acid for 1 h at room temperature, and destained again with 40% methanol and 10% acetic acid.

### 2.4. Protein Identification by Liquid Chromatography–Tandem Mass Spectrometry (LC-MS/MS) Analysis

Coomassie brilliant blue-stained protein bands from 20 μg of the two secretome batches (S1 and S2) were cut from the interface between the stacking and resolving layers of a SDS-PAGE gel (with a larger stacking layer). These were in-gel destained, reduced with DTT, alkylated with iodoacetamide, and digested with trypsin (Roche, Mannheim, Germany) overnight at 37 °C as described [47,48]. Samples were then freeze-dried in a Speed-vac, resuspended in 2% acetonitrile and 0.1% formic acid, and stored at −20 °C until nanoLC-MS/MS analysis.

Peptides were then analyzed by reversed-phase LC-electrospray ionization MS/MS (RP-LC-ESI-MS/MS) in an EASY-nLC 1000 system coupled to the Q-Exactive HF mass spectrometer through the nano-Easy spray source (Thermo Scientific, Waltham, MA, USA). Peptides were loaded first onto a Acclaim PepMap 100 trapping column (20 mm × 75 µm ID, 3 µm C18 resin with 100 Å pore size; Thermo Scientific) using buffer A (mobile phase A: 2% acetonitrile and 0.1% formic acid), and then separated and eluted on a C18 resin analytical column NTCC (150 mm × 75 µm ID, 3 µm C18 resin with 100 Å pore size; Nikkyo Technos Co., Ltd., Tokyo, Japan) with an integrated spray tip. A 150 min gradient from 5% to 35% buffer B (100% acetonitrile, 0.1% formic acid) in buffer A at a constant flow rate of 250 nL/min was used. Data acquisition was performed on a Q-Exactive HF mass spectrometer. Data were acquired using an ion spray voltage 1.8 KV and ion transfer temperature of 250 °C. All data were acquired using data-dependent acquisition (DDA) and in positive mode with Xcalibur 4.0 software. For MS^2^ scan, the top 15 most abundant precursors with charges of 2 to 4+ in MS^1^ scans were selected for higher energy collisional dissociation (HCD) fragmentation with a dynamic exclusion of 20 s. The MS^1^ scans were acquired at m/z range of 350–1600 Da with mass resolution of 60,000 and automatic gain control (AGC) target of 3E6 at a maximum ion time (ITmax) of 60 ms. The threshold to trigger MS^2^ scans was 2E3. The normalized collision energy (NCE) was 27%. The resolved fragments were scanned at mass resolution of 30,000 and AGC target value of 1E5 in an ITmax of 100 ms.

Peptide identification from raw data was carried out using Mascot v.2.6.1 search engine through the Protein Discoverer 2.2 software (Thermo Scientific). A database search was performed against *Candida* Genome Database (CGD) Assembly 21 (6221 sequences). The following parameters were used for the searches: tryptic cleavage after Arg and Lys, up to two missed cleavage sites allowed, and tolerances of 10 ppm for precursor ions and 0.02 Da for MS/MS fragment ion. The searches were performed allowing optional methionine oxidation and methionine loss plus acetyl protein *N*-terminal, and fixed carbamidomethylation of cysteine. Search against decoy database (integrated decoy approach) was used to calculate FDR. The Mascot scores were adjusted by the Mascot percolator algorithm. The acceptance criteria for protein identification were an FDR < 1% and at least one peptide identified with high confidence (confidence interval, CI > 95%). Only proteins present in both secretome batches (S1 and S2) and identified with at least two peptides were selected for further analysis. The mass spectrometry proteomics data have been deposited to the ProteomeXchange Consortium via the PRIDE partner repository with the dataset identifier PXD013933.

### 2.5. Indirect Enzyme-Linked Immunosorbent Assay (ELISA)

Indirect ELISA for measurement of serum levels of IgG antibodies to the *C. albicans* secretome was performed as described previously [49] with some modifications. Wells of microtiter plates (Maxisorp; Nunc, Roskilde, Denmark) were coated with 100 µL of 5 µg/mL *C. albicans* hyphal secreted proteins or cytoplasmic proteins (prepared as reported in [50]) in 0.1 M carbonate buffer pH 9.6 overnight at 4 °C, and then washed three times with 300 µL of washing buffer (PBS containing 0.05% Tween-20). Wells were then blocked with 300 µL of PBS containing 1% bovine serum albumin (BSA) for 2 h at 37 °C. These were washed three times with 300 µL of washing buffer, and then incubated with 100 µL of serial dilutions (from 1:250 to 1:1 024 000) of the different serum pools (ncIC, cIC and non-IC) in assay buffer (PBS containing 0.1% BSA and 0.05% Tween-20) for 2 h at 37 °C. After this, wells were washed six times with 300 µL of washing buffer, and then incubated with 100 µL of horseradish peroxidase (HRP)-labelled anti-human IgG antibodies (GE Healthcare) at a dilution of 1:3000 in assay buffer at 37 °C for 1 h. Wells were rinsed three times with 300 µL of washing buffer and once with 300 µL of PBS. They were developed with 100 µL of 0.04% o-phenylenediamine dihydrochloride (OPD, Sigma, St. Louis, MO) in 0.05 M phosphate-citrate buffer, pH 5.0, containing 0.04% hydrogen peroxide. The reaction was stopped with 100 µL of 3 N sulfuric acid. The absorbance was measured in a microplate reader at 490 nm (Bio-Rad, Hercules, CA, USA). The IgG antibody titer was calculated as the inverse of the highest dilution at which the absorbance was twofold greater than the background.

### 2.6. Immunoproteomic Analysis or SERPA

SERPA was carried out as reported previously [33,51] with some modifications. In brief, 100 µg of *C. albicans* hyphal secreted proteins were actively rehydrated in Immobiline pH 3 to 11 nonlinear (NL) gradient DryStrips (7 cm long, GE Healthcare). Isoelectric focusing (IEF) was performed on IPGphor device (GE Healthcare) using the following program for analytical gels: 30 V for 3.5 h, 60 V for 3.5 h, 60–300 V for 3 h, 300–600 V for 4 h, 600–1000 V for 4 h, 1000–8000 V for 4 h, and 8000 V for 6 h. IPG strips were reduced with balancing solution (50 mM Tris-HCl, pH 6.5, 6 M urea, 30% glycerol, 2% SDS) containing 2% DTT for 30 min, and then alkylated with balancing solution containing 2.5% iodoacetamide for 30 min in dark. The second dimension (SDS-PAGE) was carried out using a 10% polyacrylamide gel. After separating the secreted proteins by 2-DE, they were transferred to a nitrocellulose membrane (GE Healthcare) at 100 V for 1 h. Blots were then stained with Sypro Ruby protein blot stain (BioRad) and scanned using Typhon scanner (GE Healthcare). They were blocked with 5% skim milk in PBS at 4 °C overnight. Blots were rinsed with PBS and incubated with serum pools (ncIC, cIC and non-IC) at three sequential dilutions 1:500, 1:250 and 1:100 in TPBS (PBS containing 0.1% Tween-20) with 0.1% skim milk for 2 h. After 4 washing steps (20 min each) with TPBS, blots were incubated with IRDye 800 W conjugated goat anti-human IgG antibody (LI-COR Biosciences, Bad Homburg, Germany) at a dilution of 1:5000 for 1 h. After 4 washing steps with TPBS (15 min each), Odyssey system was used to detect the fluorescence signals.

For identification of immunogenic protein spots, 190 µg of protein were separated by 2-DE as described above using a slightly modified IEF program: 30 V for 3.5 h, 60 V for 3.5 h, 60–300 V for 3 h, 300–600 V for 4 h, 600–1000 V for 4 h, 1000–8000 V for 4 h, and 8000 V for 9.5 h. Preparative 2-DE gels were stained with colloidal Coomassie blue as reported [51]. Briefly, gels were fixed with 50% methanol and 2% phosphoric acid for 30 min, and then washed with water twice for 10 min each. Gels were equilibrated with 33% methanol, 3% phosphoric acid and 17% ammonium sulfate for 40 min, and 6.6% Coomassie brilliant blue G-250 in methanol was added to reach 0.066% of dye. After overnight incubation, excess dye was eliminated with successive washes with water.

Immunogenic protein spots of interest were manually excised from the preparative 2-DE gels, in-gel destained, reduced, alkylated and digested as described above. These were identified by MALDI-TOF MS analyses on a 4800 Plus Proteomics Analyzer MALDI-TOF/TOF mass spectrometer (AB Sciex, Framingham, MA) as reported previously [47]. The MALDI-TOF operated in positive reflector mode with an accelerating voltage of 20,000 V. All mass spectra were internally calibrated using peptides from the auto-digestion of trypsin. Database searches of MS data for protein identification were carried out using Mascot 2.3 (www.matrixscience.com; accessed on 15 May 2019) through the software Global Protein Server v3.6 (AB Sciex) using CGD (assembly 22; 12,421 sequences; 6,015,970 residues). Search parameters were carbamidomethyl cysteine as fixed modification; oxidized methionine as variable modification; peptide mass tolerance of 100 ppm; and one missed trypsin cleavage site. The probability scores were greater than the score fixed by Mascot as significant with a *p*-value < 0.05. For the identification of the proteins from the upper left zone of the preparative 2-DE gels, four bands were cut, destained, reduced, alkylated and in-gel digested with trypsin, and then identified by LC-MS/MS analysis as detailed above.

### 2.7. Bioinformatic Analysis

NSAF (normalized spectral abundance factor) was calculated for each LC-MS/MS-identified protein [52]. CGD was the main database used for the functional protein classification. Signal peptides were predicted using SignalP4.1 software (www.cbs.dtu.dk/services/SignalP/; accessed on 15 May 2019). The presence of a signal peptide was confirmed in CGD database. Glycosylation sites were predicted using NetNGlyc 1.0 server prediction program (which examines the sequence context of Asn-Xaa-Ser/Thr) and NetNGlyc 4.0 server prediction software (which produces predictions of the type GalNac *O*-glycosylation). Venn diagrams were performed using the Venny 2.1 tool (bioinfogp.cnb.csic.es/tools/venny/; accessed on 15 May 2019). Gene ontology (GO) Term Finder software (www.candidagenome.org/cgi-bin/GO/goTermFinder; accessed on 15 May 2019) was used to search for enriched GO terms in the input list of the identified *C. albicans* gene products compared with the genes from the whole *C. albicans* genome at the CGD.

Antibody titers were log_2_-transformed to approximate normal distribution prior to data analysis. The differences in the mean log^2^ IgG antibody titers among the study groups were assessed using one-way analysis of variance (ANOVA) with Tukey’s multiple comparison correction. *p*-Value less than 0.05 was considered significant (two-sided).

## 3. Results

### 3.1. Isolation of the C. albicans Hyphal Secretome

To set up the best conditions for the isolation of *C. albicans* hyphal secreted proteins, yeast cells were grown for 18 h in two different media (salt medium+GlcNac and Lee medium pH 6.7). Hyphal cell forms were observed in both media. Cell integrity measurements by propidium iodide revealed ~3% of cell lysis in salt medium+GlcNac, and ~7% in Lee medium, pH 6.7 (Figure S1A). Similar hyphal secreted protein yield was obtained in salt medium+GlcNac (17.1 µg per 500 mL) and Lee medium (16.8 µg per 500 mL). However, hyphal secreted protein patterns were different in both media (Figure S1B). Preliminary LC-MS/MS analysis showed a slightly higher number of identified proteins in Lee medium than in salt medium+GlcNac (115 vs. 100 proteins). A total of 43 proteins were common to both media (Figure S1C). Despite slightly higher percentage of cell lysis, bioinformatic analysis using SignalP 4.1 highlighted higher percentage of secreted proteins with signal peptide in Lee medium than in salt medium+GlcNac (23.4% vs. 2%; Table S2). Because of the higher number of proteins with signal peptide, Lee medium was used for secretome extraction.

After medium selection, the protocol was further optimized by reducing shaking conditions of cell growth and removing the centrifugation break in order to obtain minimal cell lysis. Ten fluorescence images were taken before obtaining each secretome to estimate the cell lysis percentage. This protocol reduced lysis to 1–2% (Figure S1D). Secreted protein patterns were similar among different replicates (*n* = 10; Figure S2A). These were batched in two pools with five replicates each (S1 and S2), and used for the different analyses.

### 3.2. Gel-Free LC-MS/MS Analysis of the C. albicans Hyphal Secretome

**Figure 1:**
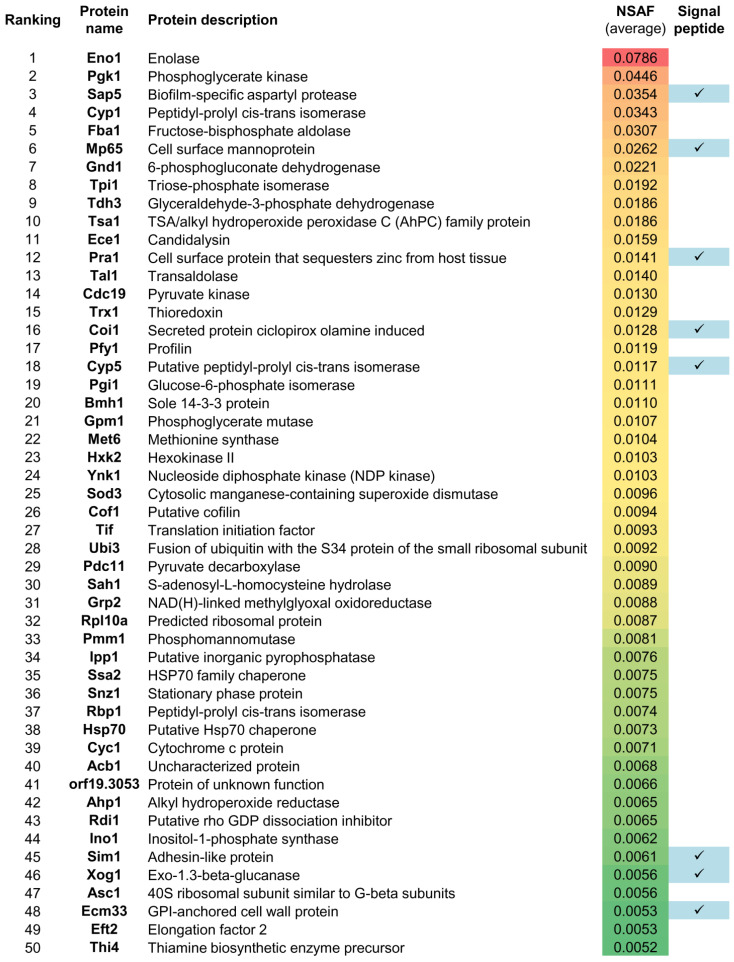
Protein ranking of the 50 most abundant proteins according to their average NSAF that were identified in the two secretome batches (S1 and S2) with at least two peptides by the gel-free LC-MS/MS analysis.

Hyphal secreted proteins in Lee medium from S1 and S2 were in-gel digested and further analyzed by LC-MS/MS. A total of 301 proteins were identified in both replicate batches with at least 2 peptides (Table S3). NSAF was used to rank proteins regarding their relative abundance (with values from 0 to 1). NSAF correlation analysis between the proteins identified in S1 and S2 showed that both secretome batches were very similar to each other in terms of protein relative abundance (Figure S2B). The 50 most abundant identified proteins from both secretome batches are shown in Figure 1.

**Figure 2:**
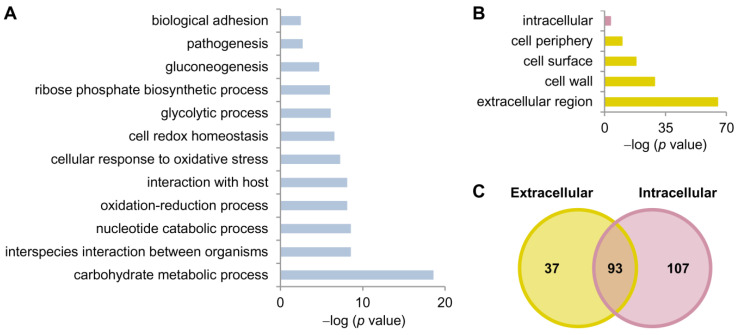
GO analysis of the proteins identified in both secretome batches (S1 and S2) with at least 2 peptides. (**A**) GO enrichment in biological process of the secreted proteins. (**B**) GO enrichment in cellular component of the secreted proteins. (**C**) Venn diagram showing the number of proteins annotated as extracellular and/or intracellular locations.

GO enrichment analysis of the identified hyphal secreted proteins highlighted that the most significantly represented biological process was carbohydrate metabolism (including proteins involved in glycolysis and cell wall polymer metabolism). Interaction with the host, cellular response to oxidative stress and pathogenesis were other enriched biological processes found in the identified proteins (Figure 2A). Proteins from the extracellular part of the cell were enriched in the secretome batches (Figure 2B). A total of 37 proteins were annotated to be present only in the extracellular region, 107 proteins were annotated only to the cytoplasmic lumen, and 93 proteins were annotated to both localizations (Figure 2C and Table 1). As expected, 43% of the proteins annotated in the extracellular region presented signal peptide, while 11% of the proteins annotated in both locations had signal peptide. Only four proteins annotated to the intracellular region were predicted to have signal peptide; these corresponded to uncharacterized ORFs. A higher percentage of immunogenic proteins were identified among those located in the extracellular region (27%) or in both cellular locations (31%) than among those located intracellularly (6.5%; Table 1).

### 3.3. Serologic Responses to the C. albicans Hyphal Secretome in ncIC, cIC and Non-IC Patients

**Figure 3:**
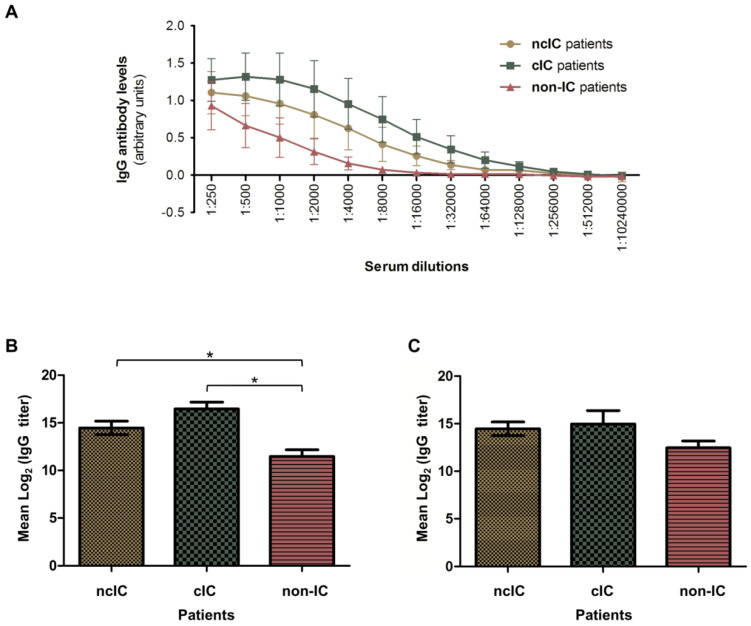
ELISA measurements of IgG antibodies against both *C. albicans* hyphal secretome batches (S1 and S2) using serum pools from ncIC, cIC and non-IC patients. (**A**) IgG levels to S1 and S2 in the three serum pools at different dilutions. (**B**) Serum titers of IgG antibodies to the *C. albicans* hyphal secretome in ncIC, cIC and non-IC patients. * *p*-value < 0.05. (**C**) Serum titers of IgG antibodies to the *C. albicans* hyphal cytoplasmic proteome in ncIC, cIC and non-IC patients.

To characterize the IgG antibody-reactivity patterns to the *C. albicans* hyphal secretome, three different pools of serum samples from 12 ncIC, 11 cIC and 11 non-IC patients were compared. Serum IgG antibody levels to both *C. albicans* hyphal secretome batches (S1 and S2) were measured by indirect ELISA. ncIC and cIC patients had higher IgG antibody levels to the *C. albicans* hyphal secretome than non-IC patients (Figure 3A). Interestingly, cIC patients showed higher, but not statistically significant, serum IgG antibody levels to *C. albicans* hyphal secreted proteins than ncIC patients. The mean IgG antibody titers against the *C. albicans* hyphal secretome were significantly higher in ncIC and cIC patients (14.5 and 16.5, respectively) than in non-IC patients (11.5, *p* = 0.01; Figure 3B). In contrast, IgG antibody titers against *C. albicans* hyphal cytoplasmic extract were not significantly higher in ncIC and cIC patients when compared to non-IC patients (*p* = 0.2, Figure 3C).

**Figure 4:**
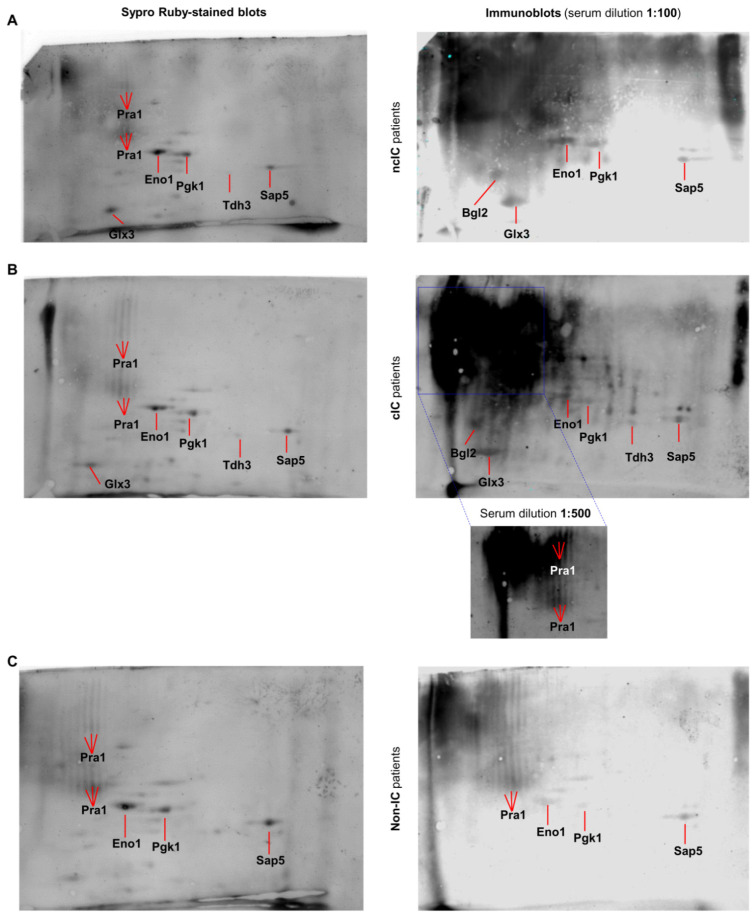
Representative Sypro Ruby-stained blots (left) and immunoblots (right) using sera from (**A**) ncIC, (**B**) cIC, and (**C**) non-IC patients at a dilution of 1:100. In (**B**), a part of the 2-D immunoblot with a lower dilution of sera (1:500) showing defined spots instead of the smear. The immunoreactive proteins identified by MALDI-TOF are labeled.

These results prompted us to analyze the serologic responses from the different patient groups using SERPA. Different immunoreactivity patterns were found in these groups (Figure 4). *C. albicans* hyphal secreted proteins showed higher immunoreactivity in ncIC and cIC patients than in non-IC patients, in line with ELISA data. Seven immunogenic secreted proteins from *C. albicans* hyphae involved in cell wall remodeling (Bgl2), metabolism (Eno1, Glx3, Pgk1 and Tdh3), and host interaction (Pra1 and Sap5) were identified (Table 2 and Figure S3). Bgl2 and Glx3 were recognized by serum pools from ncIC and cIC patients but not from non-IC patients. Eno1, Pgk1, Pra1 were more immunoreactive with sera from ncIC and cIC patients than from non-IC patients. Sap5 was more immunoreactive in cIC patients than in ncIC and non-IC patients, in which showed similar immunoreactivity levels. Pra1 was the most immunoreactive protein (especially in cIC patients). Tdh3 was exclusively immunorecognized by sera from cIC patients.

High levels of immunoreactivity were observed in hyphal secreted proteins located on the upper-left high-molecular-weight (HMW)/acidic corner of the 2-D immunoblots, particularly when these were incubated with sera from ncIC and cIC patients. Four protein bands were excised from this zone of the 2-DE gel and identified by LC-MS/MS (Table 3 and Table S4). The majority of the identified proteins were associated with the cell wall (Tos1, Ecm33, Sim1, Sun41, Cht3 and Mp65) and the cell surface or extracellular medium (Rbt4 and Pra1). The theoretical molecular weights of the identified proteins were lower than those of this zone of the 2-DE gel (except for Cht3 and Scw11), which is in accordance with the high degree of glycosylation of many cell wall proteins. The more abundant identified proteins had at least one predicted glycosylation site (Table 3).

## 4. Discussion

### 4.1. The C. albicans Hyphal Secretome Comprises Proteins Involved in Interaction with Host and Immunogenic Proteins

Lee medium was selected for *C. albicans* cell growth mainly due to the identification of more proteins with signal peptide than in the other medium assessed. This medium was previously used to induce hyphal morphology in *C. albicans* [45,55,56]. GO enrichment analysis revealed that the identified extracellular proteins were mainly involved in interaction with the host (such as pathogenesis, cellular response to oxidative stress, induction by symbiont of host defense response, and adhesion to the host). Among those, we identified proteins from the Sap family (Sap5, Sap4, Sap10 and Sap8), which are implicated in degradation of host proteins [57], Als1 and Xog1, which are involved in cell adherence and biofilm formation [58], and Ecm33, which is required for cell wall integrity, morphogenesis, virulence, as well as response to temperature, osmotic and oxidative stress in *C. albicans* [59,60,61,62,63].

Different proteins located at the cell wall were also identified, such as some GPI-anchored (Ecm33, Pga4, Rhd3, Utr2) as well as non-covalently attached (Kre9, Bgl2, Eng1, Mp65, Cht1, Cht2) cell wall proteins [64,65,66]. The presence of cell wall proteins in the *C. albicans* secretome was previously noticed in other works [33,64,67]. This is consistent with the fact that some of them may be detached from the cell wall during cell growth, or fail to incorporate in the first place. Only 12% of the genes for the identified proteins encoded for signal peptide. However, several proteins were described as being located both intra- and extracellularly, suggesting that a great number of proteins were secreted by non-canonical mechanisms. These proteins may be secreted inside EVs [35,38,39,43,68]. When comparing the proteins identified in this study with those reported in EVs and EVs-free secretome from *C. albicans* [39], we observe that 16 proteins (Bgl2, Cht1, Cht3, Coi1, Cyp5, Ecm33, Eng1, Mp65, orf19.4952.1, Pga4, Phr2, Rbe1, Rhd3, Sim1, Utr2, and Xog1) were common to all conditions. Only 4 proteins (Asc1, Hex1, Sap10, and Sap8) were common between our secretome and the EV-free secretome described previously. Interestingly, other 16 proteins (Cyp1, Eft2, Eno1, Gpm1, Hsp70, Met6, Mnt1, Pdc11, Pgk1, Por1, Rho1, Sah1, Ssa2, Tal1, Tdh3, and Ykt6) were common only between EVs secretome [35] and the secretome described here. Some of the identified proteins are moonlighting proteins, such as Tdh3, which is a well-known housekeeping enzyme found also at the cell surface of *C. albicans* and to be immunogenic [69], or Eno1 and Eft2, which can bind serum proteins [40,70], among others.

We found that 190 out of the 301 identified proteins were already described in earlier studies on the *C. albicans* hyphal secretome [33,67,71] (Table S5). Out of the 111 remaining identified proteins, 4 and 48 were already reported in a gel-based proteomic work [33], and in a recent study showing the comparison of the proteomic composition of planktonic and biofilm EVs from *C. albicans* (where the planktonic cells were in the hyphal form) [43], respectively (Figure S4). These observations suggest that several proteins identified in this study were secreted by non-conventional pathways of secretion [38,39,43]. These dissimilarities in the composition of the *C. albicans* hyphal secretome may be attributed to differences in cell growth media, time and temperature of incubation, and extraction protocol (Figure S1), as well as the type of mass spectrometers used for protein identification, among others, as pointed out previously [35]. With the MS technology evolving so quickly and its detection sensitivity increasing, more proteins can consequently be identified in the fungal secretomes. Some of the proteins identified in the hyphal secretome were previously described in the *C. albicans* surfome or surfaceome (such as Cip1, Cmd1, Egd1, Hnt1, Hsp12, Mdg1, orf19.5943.1, orf19.7196 and orf19.7368) [72,73,74,75,76] (Table S6). As these proteins are non-covalently attached to the cell surface, they may have detached during the experimental procedures of this study, or be located at the cell surface in their transition to the extracellular environment.

The proteomic analysis performed on the *C. albicans* hyphal secretome enabled the identification of 47 proteins previously characterized as immunogenic proteins (Table 4). Most of them did not have signal peptide and were annotated in the extra- and intracellular compartments.

### 4.2. The C. albicans Hyphal Immunosecretome Recognized by IgG Antibodies Differs between IC and Non-IC Patients

Serum IgG antibody levels to the *C. albicans* hyphal secretome were higher in ncIC and cIC patients than in non-IC patients, indicating that IC patients mounted stronger IgG antibody responses to *C. albicans* hyphal secreted proteins than non-IC patients. These results are in line with earlier studies [24,28,29,49,78]. The slightly, but not significantly, higher immunoreactivity of this subproteome in cIC patients than in ncIC patients could be attributed, at least in part, to the easy access of *C. albicans* cells from the contaminated catheters to bloodstream in cIC, as well as to the lower amount of *C. albicans* cells into the bloodstream in ncIC because IC was associated with candidemia (ncIC and cIC) and/or deep-seated candidiasis (ncIC), resulting in lower IgG antibody levels to the *C. albicans* hyphal secretome. Differences in antibody-reactivity patterns between ncIC and cIC were more pronounced in SERPA assays. The *C. albicans* secretome encompasses proteins with contrasting binding properties and glycosylation levels (such as, classical adhesins and moonlighting proteins [35,39]), which can be bound to the microplate wells in distinct ways. These observations could explain at least in part the differences encountered between ELISA and SERPA results. It is therefore conceivable that increasing concentrations of immobilized proteins from these complex and heterogeneous mixtures on the microplate wells, as assayed by ELISA, may unmask differences in the serological responses to the *C. albicans* secretome between both IC groups, as well as to *C. albicans* cytoplasmic extracts among the study groups.

### 4.3. IgG Antibodies to Bgl2, Eno1, Pgk1, Glx3, Pra1, Sap5 and Tdh3 Are IC Diagnostic Biomarker Candidates

We identified seven highly immunoreactive *C. albicans* secreted proteins (Bgl2, Eno1, Pgk1, Glx3, Sap5, Pra1 and Tdh3). IgG antibody titers to Bgl2, Eno1, Pgk1 and Glx3 enabled the discrimination of IC (ncIC and cIC) patients from non-IC patients, whereas IgG antibodies to Sap5, Pra1 and Tdh3 allowed the discrimination between cIC and non-IC patients, but not between ncIC and non-IC patients. Although more than one protein may be in a single 2-DE spot [82], previous studies showing that recombinant forms of these identified *C. albicans* proteins can bind antibodies mounted by IC patients or even trigger antibody responses in animal models during IC [27,39,54,78,83,84,85,86,87,88] support the notion that they are bona fide *C. albicans* immunogenic proteins.

Bgl2 was identified by LC-MS/MS only at a lower ranking position, indicating a lower abundance than the other secreted proteins (Nr 55, NSAF 0.0045). At the same time Bgl2 elicited higher IgG antibody levels in both IC groups than in the control group, which did not show immunoreactivity to this secreted protein. Anti-Bgl2 IgG antibodies were previously reported as a biomarker candidate for IC diagnosis [24]. Bgl2 is a 1,3-beta-glucosyltransferase localized in the cell wall active on β-1,3 glucan molecules, resulting in a β-1,3-glucan chain elongated with a β-1,6 glucan at the transfer site [89]. This protein also contributes to the delivery and accumulation of glucan for biofilm matrix building [90]. It was previously observed in the *C. albicans* yeast and hyphal secretomes [33,39,64,67,71].

Eno1 and Pgk1 were at the highest ranking positions (1 and 2 respectively), indicating that they are highly abundant in the extracellular medium of *C. albicans* hyphal cells, and triggered IgG antibody responses that also enabled the differentiation between IC and non-IC patients in line with previous studies [24,28,29,78]. Eno1 is a multifunctional protein with key roles in the glycolytic pathway, colonization of mammalian intestinal epithelium [91], and binding to human plasminogen [40,92]. Pathogens recruit the host plasminogen to increase their invasive capacity [92,93]. Pgk1 is also a moonlighting protein involved in the glycolysis and binding to plasminogen [94]. Both proteins have been found both in the cytoplasm and in the cell wall [56,95].

Glx3 was detected by LC-MS/MS but it was not included in the list of identified proteins due to the statistical filters applied. However, it was also abundant in the preparative 2-DE gel. Interestingly, it was found to be immunoreactive in the IC groups, but it did not display immunoreactivity in the control group. Glx3 is a glyoxalase that converts methylglyoxal to *D*-lactate [96]. Most significantly, it is a very abundant protein in the biofilm extracellular matrix [97]. *C. albicans glx3Δ* mutant showed impaired growth on media with glycerol. The mutant has impaired filamentation and biofilm formation [97]. In addition to being present in biofilm matrix, it has also been found in *C. albicans* yeast and hyphal secretomes [33]. Glx3 was previously described as an immunogenic protein recognized by sera from IC patients [25,27,28,29,78].

Sap5 is part of the large family of secreted aspartyl proteases, which are associated with *C. albicans* virulence. This protein was ranked as third in LC-MS/MS identification. Sap family play important roles in degradation of proteins, and formation of biofilms associated with bloodstream infections caused by *C. albicans* [57,98]. This protein has been found in the *C. albicans* hyphal secretome [33,67], and to be an immunogenic protein [54]. We observed no significant differences in their IgG antibody-reactivity levels between ncIC and non-IC patients, in keeping with an earlier study [54].

Pra1 is a cell-wall associated protein [58]. This hyphal secreted protein was ranked in twelfth position according to its NSAF and was found to be highly abundant in the preparative 2-DE gel with characteristics of glycosylation. We also observed that was more immunoreactive in cIC patients than in non-IC patients. However, their elicited IgG antibodies did not enable the clear differentiation between ncIC and non-IC patients. A monoclonal antibody directed towards Pra1 has shown to confer protection [81]. *C. albicans* secretes Pra1 to sequester zinc from host cells [36]. Furthermore, Pra1 binds and complexes the complement molecules C3 and blocks C3 conversion by the host C3 convertases, and blocks the C3a antifungal activity [99].

Tdh3 was ranked in the ninth position of abundance, and it was also detectable in the preparative 2-DE gel. It has been found be immunogenic [25,29,50,69], and located at the *C. albicans* cell wall proteomes [56,69] and yeast and hyphal secretomes [33]. Although their IgG antibody-reactivity levels were low, these allowed the differentiation of cIC patients from non-IC patients, but not between ncIC and non-IC patients.

The area corresponding to the more acidic and higher molecular mass proteins on 2-DE gels was also enriched for highly glycosylated proteins. In spite of being difficult to correlate the antigenicity levels to a specific identifiable protein due to the high background, many of the identified proteins in this area of the 2-DE gel were predicted at least with one bioinformatic tool to have one glycosylation site (Table 3) or described previously to be glycosylated. In particular, Mp65, Sun41, Cht3 and Ecm3 are proteins located at the cell surface, and previously described to be glycosylated [79,100,101]. Mp65 was also found to be more abundantly released in hyphal forms of *C. albicans* and to stimulate cell-mediated immune responses [102].

*C. albicans* glycosylated cell wall proteins are covalently attached to structural polysaccharides in two ways. Glycophosphatidylinositol (GPI)-anchored proteins, which are linked to β-1,6-glucan through a GPI remnant, and Pir proteins, which are directly linked to β-1,3-glucan [58]. Glycosylated proteins can carry different types of antigenic epitopes. Some oligosaccharides can also be antigenic. Another type is glycopeptide epitopes defined by antibodies which recognize specific oligosaccharide structures and adjacent amino acid residues. There are peptidic epitopes, which represent either relatively short sequences of the polypeptide chains or include amino acid residues brought into proximity due to the secondary structure of the proteins [103]. Recently, the role of glycosylation in the recognition of protein antigens by antibodies was studied. Luo and co-workers studied the immunoreactivity of Sap6 on the glycosylation status and found that anti-Sap6 antibody signal was drastically reduced after deglycosylation. Furthermore, they observed that Mp65 and other cell wall proteins identified in this upper-left HMW/acidic corner of 2-DE gels showed reduced antibody recognition by sera from candidemia patients after deglycosylation [33].

## 5. Conclusions

This study provides new insight into the *C. albicans* hyphal secretome and serological response to it in ncIC, cIC and non-IC patients. Higher IgG antibody levels to C. *albicans* hyphal secretome are mounted by cIC group than by ncIC group, as well as by both IC groups than by non-IC group. Our results have also highlighted that C. *albicans* hyphal secreted Bgl2, Eno1, Glx3, Sap5, Pgk1, Pra1 and Tdh3 have immunogenic properties, and that serum IgG antibodies to C. *albicans* Bgl2, Eno1, Pgk1 and Glx3 are diagnostic biomarker candidates for IC. Future studies should be aimed to validate these candidates in larger patient cohorts.

## Figures and Tables

**Table 1:** List of the identified proteins in both secretome batches (S1 and S2) with at least two peptides classified regarding their cellular location *^a^*.

Cellular Location	No. of Identified Proteins	Protein Names
Extracellular region, cell surface, cell wall, and cell periphery	37	Ade8, **Als1**, **Bgl2**, Cht1, Cht3, Cip1, Coi1, **Ece1**, **Ecm33**, Gcy1, **Grp2**, Hex1, Kre9, Mal2, Mdg1, Met15, **Mp65**, Nit3, Ofr1, Orf19.1394, Orf19.3053, Orf19.6809, Orf19.6867, Orf19.7322, Pga12, **Pga4**, **Pra1**, Pst2, Rbe1, Rbp1, Rhd3, **Sah1**, Sap10, Sap8, **Slk19**, Sol3, Xyl2
Intracellular region	107	**Ade17**, Aha1, Ams1, Anb1, Arf2, Arg1, Arg3, Arg4, Aro2, **Asc1**, Bfr1, Bud7, Cit1, Cmd1, Cpr6, Crm1, Cyc1, Dtd2, Ecm4, Egd1, Eif4e, Erg10, Erg20, Etr1, Fbp1, Fum11, Gcv3, Grs1, Grx3, Het1, His1, Hom2, Hta3, **Hxk2**, Idh1, Kex1, Krs1, Lat1, Lsc1, Lys21, Mca1, Mcr1, **Mdh1**, Mmd1, Mrf1, Mxr1, Npt1, Ntf2, Orf19.1355, Orf19.1448.1, Orf19.1738.1, Orf19.1815, Orf19.2930, Orf19.3319, Orf19.3681, Orf19.3932, Orf19.4150, Orf19.4382, Orf19.4898, Orf19.518, Orf19.5322, Orf19.5943.1, Orf19.5961, Orf19.6559, Orf19.6596, Orf19.6701, Orf19.6872, Orf19.7152, **Orf19.7196**, **Orf19.7214**, Orf19.7297, Orf19.7330, Orf19.7368, Orf19.7404, Orf19.7531, Orf19.7578, Orf19.86, Orf19.904, Pol30, Rdi1, Rnr21, Rpl10a, Rpl12, Rpl30, Rpp0, Rps12, Rps19a, Rps21b, Rps22a, Sbp1, Sec14, Skp1, Sno1, Snz1, Sod2, Sod3, Sub2, Sui1, Tfs1, Thi4, **Tif1**, Tub1, Tub2, Tup1, Uba1, Yhb1, Ykt6
Both locations	93	Aat21, Abp1, Acb1, **Aco1**, Ahp1, Ape3, **Atp1**, **Atp2**, Bmh1, Cam1, Cat1, **Cdc19**, Cdc3, Cof1, Cyp1, Cyp5, **Eft2**, Egd2, Emp24, Eng1, **Eno1**, **Fba1**, Fdh1, Gdh3, Gnd1, Gpd2, Gph1, **Gpm1**, Gsp1, **Hem13**, Hsp12, **Hsp70**, **Hsp90**, **Ino1**, **Ipp1**, Kel1, Leu2, Lpd1, Lsp1, **Mdh1-1**, **Met6**, Mir1, Mlc1, Mnt1, Orf19.1085, Orf19.1862, Orf19.1946, Orf19.3915, Orf19.4395, Orf19.4597, Orf19.5342, **Pdc11**, Pet9, Pfy1, **Pgi1**, **Pgk1**, Phr2, Pin3, Pmm1, **Por1**, **Prx1**, Pst3, Rho1, Rib3, Rpl14, Rpl6, Rps20, Sam2, Sap4, Sap5, Sec4, Sim1, Smt3, Sod1, Spe3, **Ssa2**, **Ssb1**, **Tal1**, **Tdh3**, Tma19, **Tpi1**, Tpm2, Trr1, Trx1, **Tsa1**, Ttr1, Ubi3, Ugp1, **Utr2**, Vps21, **Xog1**, Ynk1, Ypt1

*^a^* CGD and its GO tool were used for this analysis. In bold, proteins that were previously described to be immunogenic in *C. albicans* [24,25,27,33,53,54]. Proteins highlighted in grey are those that have signal peptide.

**Table 2:** Recognition patterns of *C. albicans* hyphal secreted proteins by serum pools from ncIC, cIC and non-IC patients.

Immunoreactive *C. albicans* Hyphal Secreted Proteins								IgG Antibody- Reactivity Levels *^a^*		
Protein Name	Protein Description	LC-MS/MS			MALDI-TOF-MS			ncIC	cIC	Non- IC
		No. of Peptides	Ranking According to NSAF	NSAF	No. of Matched/ Unmatched Peptides	Mascot Score	% of Sequence Coverage			
Bgl2	Cell wall 1,3-beta- glucosyltransferase	8	55	0.004	10/58	213	20	+++	+++	−
Eno1	Enolase	30	1	0.08	9/61	157	32	++++	+++	+
Glx3	Glutathione- independent glyoxalase	NI *^b^*	NI *^b^*	NI *^b^*	12/56	276	61	+++	+++	−
Pgk1	Phosphoglycerate kinase	38	2	0.04	21/47	460	48	+++	++	+
Pra1	pH regulated antigen	10	12	0.01	11/57	240	29	ND	+++++	+++
					9/59	218	16			
					10/58	229	20			
					7/61	75	8			
					7/60	115	11			
					5/63	96	7			
Sap5	Secreted aspartyl proteinase	26	3	0.03	14/54	272	39	++	+++	++
					28/43	815	60			
					39/38	1220	67			
					6/62	60	18			
Tdh3	Glyceraldehyde-3- phosphate	27	9	0.02	16/53	140	51	−	+	−

*^a^* From no (−) or less (+) to more (+++++) immunoreactivity. *ND* denotes that protein immunoreactivity was not possible to determine. *^b^ NI* means that this protein was identified by LC-MS/MS but it did not pass the statistical filters.

**Table 3:** The more abundant proteins identified by LC-MS/MS in the left upper corner of a preparative 2-DE gel of the *C. albicans* hyphal secretome (NSAF higher than 0.01).

Standard Name	Description	NetNGlyc 1.0 Server Prediction	NetNGlyc 4.0 Server Prediction	NSAF	No. Peptides	M*r*
Rbt4	Pry family protein	no	yes	0.19	11	37.4
Mp65	Cell surface mannoprotein	no	yes	0.15	13	39.3
Sun41	Cell wall glycosidase	yes	yes	0.12	11	43.7
Tos1	Protein similar to alpha agglutinin anchor subunit	no	yes	0.10	15	49.4
Ecm33	GPI-anchored cell wall protein	yes	yes	0.03	6	43.5
Cht3	Major chitinase	no	yes	0.03	7	60.0
Pra1	Cell surface protein that sequesters zinc from host tissue	yes	yes	0.02	7	33.1
Glx3	Glutathione-independent glyoxalase	yes	no	0.02	12	25.8
Sim1	Adhesin-like protein	yes	yes	0.01	9	39.4
Scw11	Cell wall protein	yes	yes	0.01	10	54.4

**Table 4:** LC-MS/MS-identified proteins that have shown to have immunogenic properties in previous studies.

Standard Name^*a*^	Description *^a^*	Cell Localization^*b*^	Signal Peptide *^a^*	References *^c^*
Aco1	Aconitase	Shared	No	[25,77]
Ade17	5-Aminoimidazole-4-carboxamide ribotide transformylase	Intracellular	No	[25]
Als1	Cell-surface adhesin	Extracellular	yes	[54]
Asc1	40S ribosomal subunit similar to G-beta subunits	Intracellular	No	[25]
Atp1	ATP synthase alpha subunit	Shared	No	[25]
Atp2	F1 beta subunit of F1F0 ATPase complex	Shared	No	[25]
Bgl2	Cell wall 1,3-beta-glucosyltransferase	Extracellular	Yes	[24]
Cdc19	Pyruvate kinase at yeast cell surface	Shared	No	[77]
Ece1	Extent of cell elongation protein	Extracellular	yes	[54]
Ecm33	GPI-anchored cell wall protein	Extracellular	yes	[54]
Eft2	Elongation factor 2	Shared	No	[77]
Eno1	Enolase	Shared	No	[50,53,78]
Fba1	Fructose-bisphosphate aldolase	Shared	No	[25]
Gnd1	6-phosphogluconate dehydrogenase	Shared	No	[29]
Gpm1	Phosphoglycerate mutase	Shared	No	[25,77]
Grp2	NAD(H)-linked methylglyoxal oxidoreductase involved in regulation of methylglyoxal and pyruvate levels	Extracellular	No	[25]
Hem13	Coproporphyrinogen III oxidase	Shared	No	[25]
Hsp70	Putative Hsp70 chaperone	Shared	No	[25,53]
Hsp90	Essential chaperone	Shared	No	[25,53]
Hxk2	Hexokinase II	Intracellular	No	[25]
Ino1	Inositol-1-phosphate synthase	Shared	No	[25]
Ipp1	Putative inorganic pyrophosphatase	Shared	No	[25]
Mdh1	Mitochondrial malate dehydrogenase	Intracellular	Yes	[25]
Mdh1-1	Predicted malate dehydrogenase precursor	Shared	No	
Met6	5-methyltetrahydropteroyltriglutamate-homocysteine methyltransferase (methionine synthase)	Shared	No	[49,77]
Mp65	Cell surface mannoprotein	Extracellular	Yes	[79,80]
Msi3	Essential HSP70 family protein	Intracellular	No	
orf19.7196	Putative vacuolar protease	Intracellular	Yes	[33]
orf19.7214	Glucan 1,3-beta-glucosidase	Intracellular	No	[54]
Pdc11	Pyruvate decarboxylase	Shared	No	[25]
Pga4	GPI-anchored cell surface protein	Extracellular	Yes	[54]
Pgi1	Glucose-6-phosphate isomerase	Shared	No	[25]
Pgk1	Phosphoglycerate kinase	Shared	No	[50,53]
Por1	Mitochondrial outer membrane porin	Shared	No	[25]
Pra1	pH regulated antigen	Extracellular	Yes	[54,81]
Prx1	Thioredoxin peroxidase	Shared	No	[33]
Sah1	S-adenosyl-L-homocysteine hydrolase	Extracellular	No	[25]
Slk19	Alkaline-induced protein of plasma membrane	Extracellular	no	[54]
Ssa2	HSP70 family chaperone	Shared	No	[25,54]
Ssb1	HSP70 family heat shock protein	Shared	No	[25,32]
Tal1	Transaldolase	Shared	No	[33]
Tdh3	NAD-linked glyceraldehyde-3-phosphate dehydrogenase	Shared	No	[25,50,69]
Tif	Translation initiation factor	Intracellular	No	[25]
Tpi1	Triose-phosphate isomerase	Shared	No	[25,32]
Tsa1	TSA/alkyl hydroperoxide peroxidase C (AhPC) family protein	Shared	No	[27]
Utr2	Putative GPI anchored cell wall glycosidase	Extracellular	yes	[54]
Xog1	Exo-1,3-beta-glucanase	Shared	Yes	[54]

*^a^* Names, description and prediction of signal peptide according to CGD. *^b^* Localization according to GO enrichment performed in CGD. “Shared” means that the protein was described intra- and extra-cellularly. “Extracellular” embraces extracellular region, cell surface, cell wall and cell periphery. *^c^* Previous studies where these proteins were shown to have immunogenic properties.

## Data Availability

The mass spectrometry proteomics data have been deposited to the ProteomeXchange Consortium via the PRIDE partner repository with the dataset identifier PXD013933.

## Supplementary Materials

The following are available online at https://www.mdpi.com/article/10.3390/jof7070501/s1, Figure S1: Isolation of the *C. albicans* hyphal secretome in two different growth media, Figure S2: *C. albicans* hyphal secretome samples isolated in Lee medium (pH 6.7), Figure S3: Coomassie blue-stained preparative 2-DE gel of the *C. albicans* hyphal secretome, Figure S4: Schematic Venn diagrams comparing *C. albicans* secreted proteins described here and in other studies, Table S1: Baseline characteristics of the patients included in this study, Table S2: *C. albicans* hyphal secreted proteins identified after cell growth in salt medium+GlcNac and Lee medium, Table S3: *C. albicans* hyphal secreted proteins identified with at least 2 peptides and present in both independent batches (S1 and S2), Table S4: *C. albicans* hyphal secreted proteins identified with at least 2 peptides in the left upper corner of the preparative 2-DE gel, Table S5: Comparison between proteins previously reported in the hyphal *C. albicans* secretome or biofilm EVs and our study, Table S6: *C. albicans* hyphal secreted proteins identified in our study that were previously described in surfome studies.

## References

[1] Sudbery P., Gow N., Berman J. (2004). The distinct morphogenic states of *Candida albicans*. Trends Microbiol..

[2] Höfs S., Mogavero S., Hube B. (2016). Interaction of *Candida albicans* with host cells: Virulence factors, host defense, escape strategies, and the microbiota. J. Microbiol..

[3] Gow N.A., Hube B. (2012). Importance of the *Candida albicans* cell wall during commensalism and infection. Curr. Opin. Microbiol..

[4] Wilson D., Naglik J.R., Hube B. (2016). The missing link between *Candida albicans* hyphal morphogenesis and host cell damage. PLoS Pathog..

[5] Pappas P.G., Lionakis M.S., Arendrup M.C., Ostrosky-Zeichner L., Kullberg B.J. (2018). Invasive candidiasis. Nat. Rev. Dis. Primers.

[6] Kullberg B.J., Arendrup M.C. (2015). Invasive candidiasis. N. Engl. J. Med..

[7] Pitarch A., Nombela C., Gil C. (2018). Diagnosis of invasive candidiasis: From gold standard methods to promising leading-edge technologies. Curr. Top. Med. Chem..

[8] Nucci M., Anaissie E. (2001). Revisiting the source of candidemia: Skin or gut?. Clin. Infect. Dis..

[9] Phua A.I.-H., Hon K.Y., Holt A., O’Callaghan M., Bihari S. (2019). *Candida* catheter-related bloodstream infection in patients on home parenteral nutrition—Rates, risk factors, outcomes, and management. Clin. Nutr. ESPEN.

[10] Magill S.S., Edwards J.R., Bamberg W., Beldavs Z.G., Dumyati G., Kainer M.A., Lynfield R., Maloney M., McAllister-Hollod L., Nadle J. (2014). Multistate point-prevalence survey of health care-associated infections. N. Engl. J. Med..

[11] Chaves F., Garnacho-Montero J., Del Pozo J.L., Bouza E., Capdevila J.A., de Cueto M., Dominguez M.A., Esteban J., Fernandez-Hidalgo N., Fernandez Sampedro M. (2018). Diagnosis and treatment of catheter-related bloodstream infection: Clinical guidelines of the Spanish Society of Infectious Diseases and Clinical Microbiology and (SEIMC) and the Spanish Society of Spanish Society of Intensive and Critical Care Medicine and Coronary Units (SEMICYUC). Med. Intensiva.

[12] Clancy C.J., Nguyen M.H. (2013). Finding the “missing 50%” of invasive candidiasis: How nonculture diagnostics will improve understanding of disease spectrum and transform patient care. Clin. Infect. Dis..

[13] Posch W., Heimdörfer D., Wilflingseder D., Lass-Flörl C. (2017). Invasive candidiasis: Future directions in non-culture based diagnosis. Expert Rev. Anti Infect. Ther..

[14] Pfaller M.A., Castanheira M. (2015). Nosocomial candidiasis: Antifungal stewardship and the importance of rapid diagnosis. Med. Mycol..

[15] Clancy C.J., Nguyen M.H. (2018). T2 magnetic resonance for the diagnosis of bloodstream infections: Charting a path forward. J. Antimicrob. Chemother..

[16] Mylonakis E., Clancy C.J., Ostrosky-Zeichner L., Garey K., Alangaden G.J., Vazquez J.A., Groeger J.S., Judson M.A., Vinagre Y.-M., Heard S.O. (2015). T2 magnetic resonance assay for the rapid diagnosis of candidemia in whole blood: A clinical trial. Clin. Infect. Dis..

[17] Neely L.A., Audeh M., Phung N.A., Min M., Suchocki A., Plourde D., Blanco M., Demas V., Skewis L.R., Anagnostou T. (2013). T2 magnetic resonance enables nanoparticle-mediated rapid detection of candidemia in whole blood. Sci. Transl. Med..

[18] Krah A., Jungblut P.R. (2004). Immunoproteomics. Methods Mol. Med..

[19] Dea-Ayuela M.A., Ubeira F.M., Pitarch A., Gil C., Martinez-Fernandez A.R., Bolas F. (2001). A comparison of antigenic peptides in muscle larvae of several *Trichinella* species by two-dimensional Western-blot analysis with monoclonal antibodies. Parasite.

[20] Jungblut P.R., Bumann D. (2002). Immunoproteome of *Helicobacter pylori*. Methods Enzymol..

[21] Pedersen S.K., Sloane A.J., Prasad S.S., Sebastian L.T., Lindner R.A., Hsu M., Robinson M., Bye P.T., Weinberger R.P., Harry J.L. (2005). An immunoproteomic approach for identification of clinical biomarkers for monitoring disease: Application to cystic fibrosis. Mol. Cell Proteomics.

[22] Thomas D.P., Pitarch A., Monteoliva L., Gil C., Lopez-Ribot J. (2006). Proteomics to study *Candida albicans* biology and pathogenicity. Infect. Disord. Drug Targets.

[23] Pitarch A., Sánchez M., Nombela C., Gil C. (2003). Analysis of the *Candida albicans* proteome. I. Strategies and applications. J. Chromatogr. B Analyt. Technol. Biomed. Life Sci..

[24] Pitarch A., Jiménez A., Nombela C., Gil C. (2006). Decoding serological response to *Candida* cell wall immunome into novel diagnostic, prognostic, and therapeutic candidates for systemic candidiasis by proteomic and bioinformatic analyses. Mol. Cell. Proteomics.

[25] Pitarch A., Abian J., Carrascal M., Sánchez M., Nombela C., Gil C. (2004). Proteomics—based identification of novel *Candida albicans* antigens for diagnosis of systemic candidiasis in patients with underlying hematological malignancies. Proteomics.

[26] Pitarch A., Nombela C., Gil C. (2006). Contributions of proteomics to diagnosis, treatment, and prevention of candidiasis. Methods Biochem. Anal..

[27] Pitarch A., Nombela C., Gil C. (2011). Prediction of the clinical outcome in invasive candidiasis patients based on molecular fingerprints of five anti-*Candida* antibodies in serum. Mol. Cell. Proteomics.

[28] Pitarch A., Nombela C., Gil C. (2014). Serum antibody signature directed against *Candida albicans* Hsp90 and enolase detects invasive candidiasis in non-neutropenic patients. J. Proteome Res..

[29] Pitarch A., Nombela C., Gil C. (2016). Seroprofiling at the *Candida albicans* protein species level unveils an accurate molecular discriminator for candidemia. J. Proteomics.

[30] Huertas B., Prieto D., Pitarch A., Gil C., Pla J., Díez-Orejas R. (2017). Serum antibody profile during colonization of the mouse gut by *Candida albicans*: Relevance for protection during systemic infection. J. Proteome Res..

[31] Pitarch A., Nombela C., Gil C., San-Blas G., Calderone R. (2008). The *Candida* immunome as a mine for clinical biomarker development for invasive candidiasis: From biomarker discovery to assay validation. Pathogenic Fungi: Insights in Molecular Biology Wymondham.

[32] Pitarch A., Diez-Orejas R., Molero G., Pardo M., Sanchez M., Gil C., Nombela C. (2001). Analysis of the serologic response to systemic *Candida albicans* infection in a murine model. Proteomics.

[33] Luo T., Krüger T., Knüpfer U., Kasper L., Wielsch N., Hube B., Kortgen A., Bauer M., Giamarellos-Bourboulis E.J., Dimopoulos G. (2016). Immunoproteomic analysis of antibody responses to extracellular proteins of *Candida albicans* revealing the importance of glycosylation for antigen recognition. J. Proteome Res..

[34] Klis F.M., Brul S. (2015). Adaptations of the secretome of *Candida albicans* in response to host-related environmental conditions. Eukaryot. Cell.

[35] Gil-Bona A., Amador-García A., Gil C., Monteoliva L. (2018). The external face of *Candida albicans*: A proteomic view of the cell surface and the extracellular environment. J. Proteomics.

[36] Citiulo F., Jacobsen I.D., Miramón P., Schild L., Brunke S., Zipfel P., Brock M., Hube B., Wilson D. (2012). *Candida albicans* scavenges host zinc via Pra1 during endothelial invasion. PLoS Pathog..

[37] Wu H., Downs D., Ghosh K., Ghosh A.K., Staib P., Monod M., Tang J. (2013). *Candida albicans* secreted aspartic proteases 4–6 induce apoptosis of epithelial cells by a novel Trojan horse mechanism. FASEB J..

[38] Nombela C., Gil C., Chaffin W.L. (2006). Non-conventional protein secretion in yeast. Trends Microbiol..

[39] Gil-Bona A., Llama-Palacios A., Parra C.M., Vivanco F., Nombela C., Monteoliva L., Gil C. (2015). Proteomics unravels extracellular vesicles as carriers of classical cytoplasmic proteins in *Candida albicans*. J. Proteome Res..

[40] Satala D., Satala G., Karkowska-Kuleta J., Bukowski M., Kluza A., Rapala-Kozik M., Kozik A. (2020). Structural insights into the interactions of candidal enolase with human vitronectin, fibronectin and plasminogen. Int. J. Mol. Sci..

[41] Pärnänen P., Sorsa T., Tervahartiala T., Nikula-Ijäs P. (2020). Isolation, characterization and regulation of moonlighting proteases from *Candida glabrata* cell wall. Microb. Pathog..

[42] Nimrichter L., De Souza M.M., Del Poeta M., Nosanchuk J.D., Joffe L., Tavares P.D.M., Rodrigues M. (2016). Extracellular vesicle-associated transitory cell wall components and their impact on the interaction of fungi with host cells. Front. Microbiol..

[43] Zarnowski R., Sanchez H., Covelli A.S., Dominguez E., Jaromin A., Bernhardt J., Mitchell K.F., Heiss C., Azadi P., Mitchell A. (2018). *Candida albicans* biofilm–induced vesicles confer drug resistance through matrix biogenesis. PLoS Biol..

[44] Yin Q.Y. (2008). Exploring the Fungal Wall Proteome by Mass Spectrometry.

[45] Lee K.L., Buckley H.R., Campbell C.C. (1975). An amino acid liquid synthetic medium for the development of mycelial and yeast forms of *Candida albicans*. Sabouraudia.

[46] Pitarch A., Nombela C., Gil C. (2016). Top-down characterization data on the speciation of the *Candida albicans* immunome in candidemia. Data Brief..

[47] Pitarch A., Nombela C., Gil C. (2009). Identification of the *Candida albicans* immunome during systemic infection by mass spectrometry. Methods Mol. Biol..

[48] Valdés I., Pitarch A., Gil C., Bermúdez A., Llorente M., Nombela C., Méndez E. (2000). Novel procedure for the identification of proteins by mass fingerprinting combining two-dimensional electrophoresis with fluorescent SYPRO Red staining. J. Mass Spectrom..

[49] Pitarch A., Nombela C., Gil C. (2007). Reliability of antibodies to *Candida* methionine synthase for diagnosis, prognosis and risk stratification in systemic candidiasis: A generic strategy for the prototype development phase of proteomic markers. Proteomics Clin. Appl..

[50] Pitarch A., Pardo M., Jimenez A., Pla J., Gil C., Sanchez M., Nombela C. (1999). Two-dimensional gel electrophoresis as analytical tool for identifying *Candida albicans* immunogenic proteins. Electrophoresis.

[51] Pitarch A., Nombela C., Gil C. (2009). Proteomic profiling of serologic response to *Candida albicans* during host-commensal and host-pathogen interactions. Methods Mol. Biol..

[52] Zybailov B., Mosley A.L., Sardiu M.E., Coleman M.K., Florens L., Washburn M.P. (2006). Statistical analysis of membrane proteome expression changes in *Saccharomyces cerevisiae*. J. Proteome Res..

[53] Martínez J.P., Gil M.L., López-Ribot J.L., Chaffin W.L. (1998). Serologic response to cell wall mannoproteins and proteins of *Candida albicans*. Clin. Microbiol. Rev..

[54] Mochon A.B., Jin Y., Kayala M.A., Wingard J.R., Clancy C.J., Nguyen M.H., Felgner P., Baldi P., Liu H. (2010). Serological profiling of a *Candida albicans* protein microarray reveals permanent host-pathogen interplay and stage-specific responses during candidemia. PLoS Pathog..

[55] Nantel A., Dignard D., Bachewich C., Harcus D., Marcil A., Bouin A.-P., Sensen C.W., Hogues H., Hoog M.V.H., Gordon P. (2002). Transcription profiling of *Candida albicans* cells undergoing the yeast-to-hyphal transition. Mol. Biol. Cell.

[56] Pitarch A., Sánchez M., Nombela C., Gil C. (2002). Sequential fractionation and two-dimensional gel analysis unravels the complexity of the dimorphic fungus *Candida albicans* cell wall proteome. Mol. Cell. Proteomics.

[57] Naglik J.R., Challacombe S.J., Hube B. (2003). *Candida albicans* secreted aspartyl proteinases in virulence and pathogenesis. Microbiol. Mol. Biol. Rev..

[58] Chaffin W.L. (2008). *Candida albicans* cell wall proteins. Microbiol. Mol. Biol. Rev..

[59] Martinez R., Monteoliva L., Diez-Orejas R., Nombela C., Gil C. (2004). The GPI-anchored protein CaEcm33p is required for cell wall integrity, morphogenesis and virulence in *Candida albicans*. Microbiology.

[60] Gil-Bona A., Reales-Calderon J.A., Giraldo C.M.P., Martinez R., Monteoliva L., Gil C. (2016). The cell wall protein Ecm33 of *Candida albicans* is involved in chronological life span, morphogenesis, cell wall regeneration, stress tolerance, and host–cell interaction. Front. Microbiol..

[61] Gil-Bona A., Monteoliva L., Gil García C. (2015). Global proteomic profiling of the secretome of *Candida albicans ecm33* cell wall mutant reveals the involvement of Ecm33 in Sap2 secretion. J. Proteome Res..

[62] Martinez-Lopez R., Park H., Myers C.L., Gil C., Filler S.G. (2006). *Candida albicans* Ecm33p is important for normal cell wall architecture and interactions with host cells. Eukaryot. Cell.

[63] Monteoliva L., Martinez R., Pitarch A., Hernaez M.L., Serna A., Nombela C., Albar J.P., Gil C. (2011). Quantitative proteome and acidic subproteome profiling of *Candida albicans* yeast-to-hypha transition. J. Proteome Res..

[64] Ene I.V., Heilmann C.J., Sorgo A.G., Walker L.A., de Koster C.G., Munro C.A., Klis F.M., Brown A.J. (2012). Carbon source-induced reprogramming of the cell wall proteome and secretome modulates the adherence and drug resistance of the fungal pathogen *Candida albicans*. Proteomics.

[65] de Groot P.W., de Boer A.D., Cunningham J., Dekker H.L., de Jong L., Hellingwerf K.J., de Koster C., Klis F.M. (2004). Proteomic analysis of *Candida albicans* cell walls reveals covalently bound carbohydrate-active enzymes and adhesins. Eukaryot. Cell.

[66] Pitarch A., Nombela C., Gil C. (2008). Cell wall fractionation for yeast and fungal. Methods Mol. Biol..

[67] Sorgo A.G., Heilmann C.J., Dekker H.L., Brul S., De Koster C.G., Klis F.M. (2010). Mass spectrometric analysis of the secretome of *Candida albicans*. Yeast.

[68] Wolf J.M., Espadas J., Luque-Garcia J.L., Reynolds T., Casadevall A. (2015). Lipid biosynthetic genes affect *Candida albicans* extracellular vesicle morphology, cargo, and immunostimulatory properties. Eukaryot. Cell.

[69] Gil-Navarro I., Gil M.L., Casanova M., O’Connor J.E., Martínez J.P., Gozalbo D. (1997). The glycolytic enzyme glyceraldehyde-3-phosphate dehydrogenase of *Candida albicans* is a surface antigen. J. Bacteriol..

[70] Karkowska-Kuleta J., Kozik A. (2014). Moonlighting proteins as virulence factors of pathogenic fungi, parasitic protozoa and multicellular parasites. Mol. Oral Microbiol..

[71] Heilmann C.J., Sorgo A.G., Mohammadi S., Sosinska G.J., De Koster C.G., Brul S., De Koning L.J., Klis F.M. (2012). Surface stress induces a conserved cell wall stress response in the pathogenic fungus *Candida albicans*. Eukaryot. Cell.

[72] Gil-Bona A., Parra-Giraldo C.M., Hernáez M.L., Reales-Calderon J.A., Solis N.V., Filler S.G., Monteoliva L., Gil C. (2015). *Candida albicans* cell shaving uncovers new proteins involved in cell wall integrity, yeast to hypha transition, stress response and host–pathogen interaction. J. Proteomics.

[73] Marin E., Parragiraldo C.M., Hernández-Haro C., Hernaez M.L., Nombela C., Monteoliva L., Gil C. (2015). *Candida albicans* shaving to profile human serum proteins on hyphal surface. Front. Microbiol..

[74] Hernáez M.L., Ximénez-Embún P., Martínez-Gomariz M., Gutiérrez-Blázquez M.D., Nombela C., Gil C. (2010). Identification of *Candida albicans* exposed surface proteins in vivo by a rapid proteomic approach. J. Proteomics.

[75] Martinez-Gomariz M., Perumal P., Mekala S., Nombela C., Chaffin W.L., Gil C. (2009). Proteomic analysis of cytoplasmic and surface proteins from yeast cells, hyphae, and biofilms of *Candida albicans*. Proteomics.

[76] Vialás V., Perumal P., Gutierrez D., Ximénez-Embún P., Nombela C., Gil C., Chaffin W.L. (2012). Cell surface shaving of *Candida albicans* biofilms, hyphae, and yeast form cells. Proteomics.

[77] Pardo M., Ward M., Pitarch A., Sanchez M., Nombela C., Blackstock W., Gil C. (2000). Cross-species identification of novel *Candida albicans* immunogenic proteins by combination of two-dimensional polyacrylamide gel electrophoresis and mass spectrometry. Electrophoresis.

[78] Pitarch A., Jiménez A., Nombela C., Gil C. (2008). Serological proteome analysis to identify systemic candidiasis patients in the intensive care unit: Analytical, diagnostic and prognostic validation of anti-*Candida* enolase antibodies on quantitative clinical platforms. Proteomics Clin. Appl..

[79] Gomez M.J., Torosantucci A., Arancia S., Maras B., Parisi L., Cassone A. (1996). Purification and biochemical characterization of a 65-kilodalton mannoprotein (MP65), a main target of anti-*Candida* cell-mediated immune responses in humans. Infect. Immun..

[80] Torosantucci A., Tumbarello M., Bromuro C., Chiani P., Posteraro B., Sanguinetti M., Cauda R., Cassone A. (2017). Antibodies against a beta-glucan-protein complex of *Candida albicans* and its potential as indicator of protective immunity in candidemic patients. Sci. Rep..

[81] Viudes A., Lazzell A., Perea S., Kirkpatrick W.R., Peman J., Patterson T.F., Martinez J.P., Lopez-Ribot J.L. (2004). The C-terminal antibody binding domain of *Candida albicans* mp58 represents a protective epitope during candidiasis. FEMS Microbiol. Lett..

[82] Yang Y., Thannhauser T.W., Li L., Zhang S. (2007). Development of an integrated approach for evaluation of 2-D gel image analysis: Impact of multiple proteins in single spots on comparative proteomics in conventional 2-D gel/MALDI workflow. Electrophoresis.

[83] Chou H., Tam M.F., Chang C.Y., Lai H.Y., Huang M.H., Chou C.T., Lee S.S., Shen H.D. (2003). Characterization of a novel *Candida albicans* 29 kDa IgE-binding protein—Purification, cDNA isolation and heterologous expression of Cand a 3. Allergy.

[84] Ardizzoni A., Posteraro B., Baschieri M.C., Bugli F., Sáez-Rosòn A., Manca L., Cacaci M., Sterbini F.P., De Waure C., Sevilla M. (2014). An antibody reactivity-based assay for diagnosis of invasive candidiasis using protein array. Int. J. Immunopathol. Pharmacol..

[85] He Z.X., Chen J., Li W., Cheng Y., Zhang H.P., Zhang L.N., Hou T.W. (2015). Serological response and diagnostic value of recombinant *Candida* cell wall protein enolase, phosphoglycerate kinase, and beta-glucosidase. Front. Microbiol..

[86] Xin H., Dziadek S., Bundle D.R., Cutler J.E. (2008). Synthetic glycopeptide vaccines combining beta-mannan and peptide epitopes induce protection against candidiasis. Proc. Natl. Acad. Sci. USA.

[87] Xin H., Cutler J.E. (2011). Vaccine and monoclonal antibody that enhance mouse resistance to candidiasis. Clin. Vaccine Immunol..

[88] Li W.Q., Hu X.C., Zhang X., Ge Y., Zhao S., Hu Y., Ashman R.B. (2011). Immunisation with the glycolytic enzyme enolase confers effective protection against *Candida albicans* infection in mice. Vaccine.

[89] Sarthy A.V., McGonigal T., Coen M., Frost D.J., Meulbroek J.A., Goldman R.C. (1997). Phenotype in *Candida albicans* of a disruption of the *BGL2* gene encoding a 1,3-beta-glucosyltransferase. Microbiology.

[90] Taff H.T., Nett J.E., Zarnowski R., Ross K.M., Sanchez H., Cain M.T., Hamaker J., Mitchell A.P., Andes D.R. (2012). A *Candida* biofilm-induced pathway for matrix glucan delivery: Implications for drug resistance. PLoS Pathog..

[91] Silva R., Padovan A.C.B., Pimenta D.C., Ferreira R.C., Da Silva C.V., Briones M.R.S. (2014). Extracellular enolase of *Candida albicans* is involved in colonization of mammalian intestinal epithelium. Front. Cell. Infect. Microbiol..

[92] Jong A.Y., Chen S.H.M., Stins M.F., Kim K.S., Tuan T.-L., Huang S.-H. (2003). Binding of *Candida albicans* enolase to plasmin(ogen) results in enhanced invasion of human brain microvascular endothelial cells. J. Med. Microbiol..

[93] Pitarch A., Nombela C., Gil C. (2006). *Candida albicans* biology and pathogenicity: Insights from proteomics. Methods Biochem. Anal..

[94] Crowe J.D., Sievwright I.K., Auld G.C., Moore N.R., Gow N.A.R., Booth N.A. (2003). *Candida albicans* binds human plasminogen: Identification of eight plasminogen-binding proteins. Mol. Microbiol..

[95] Alloush H.M., Lopez-Ribot J., Masten B.J., Chaffin W.L. (1997). 3-Phosphoglycerate kinase: A glycolytic enzyme protein present in the cell wall of *Candida albicans*. Microbiology.

[96] Hasim S., Hussin N.A., Alomar F., Bidasee K.R., Nickerson K.W., Wilson M.A. (2014). A Glutathione-independent glyoxalase of the DJ-1 superfamily plays an important role in managing metabolically generated methylglyoxal in *Candida albicans*. J. Biol. Chem..

[97] Cabello L., Gómez-Herreros E., Fernández-Pereira J., Maicas S., Martínez-Esparza M.C., De Groot P.W.J., Valentín E. (2018). Deletion of *GLX3* in *Candida albicans* affects temperature tolerance, biofilm formation and virulence. FEMS Yeast Res..

[98] Joo M.Y., Song E.S., Kee S.J., Shin J.H., Jang H.-C., Suh S.P., Ryang D.W. (2013). Expression of *SAP5* and *SAP9* in *Candida albicans* biofilms: Comparison of bloodstream isolates with isolates from other sources. Med. Mycol..

[99] Luo S., Dasari P., Reiher N., Hartmann A., Jacksch S., Wende E., Barz D., Niemiec M.J., Jacobsen I., Beyersdorf N. (2018). The secreted *Candida albicans* protein Pra1 disrupts host defense by broadly targeting and blocking complement C3 and C3 activation fragments. Mol. Immunol..

[100] Hiller E., Heine S., Brunner H., Rupp S. (2007). *Candida albicans* Sun41p, a putative glycosidase, is involved in morphogenesis, cell wall biogenesis, and biofilm formation. Eukaryot. Cell.

[101] McCreath K.J., Specht C.A., Robbins P.W. (1995). Molecular cloning and characterization of chitinase genes from *Candida albicans*. Proc. Natl. Acad. Sci. USA.

[102] Bromuro C., Torosantucci A., Gomez M., Urbani F., Cassone A. (1994). Differential release of an immunodominant 65 kDa mannoprotein antigen from yeast and mycelial forms of *Candida albicans*. Med. Mycol..

[103] Lisowska E. (2002). The role of glycosylation in protein antigenic properties. Cell. Mol. Life Sci..

