# Supplementary material for: Mass Spectrometry-Based Proteomic and Immunoproteomic Analyses of the Candida albicans Hyphal Secretome Reveal Diagnostic Biomarker Candidates for Invasive Candidiasis
Source: J Fungi (Basel). 2021 Jun 23;7(7):501. doi: 10.3390/jof7070501 (PMC8306665; doi:10.3390/jof7070501)
Supplement: Supplementary file 1 [file jof-07-00501-s001.zip › Supplemental/Figures S1-S4.pdf]

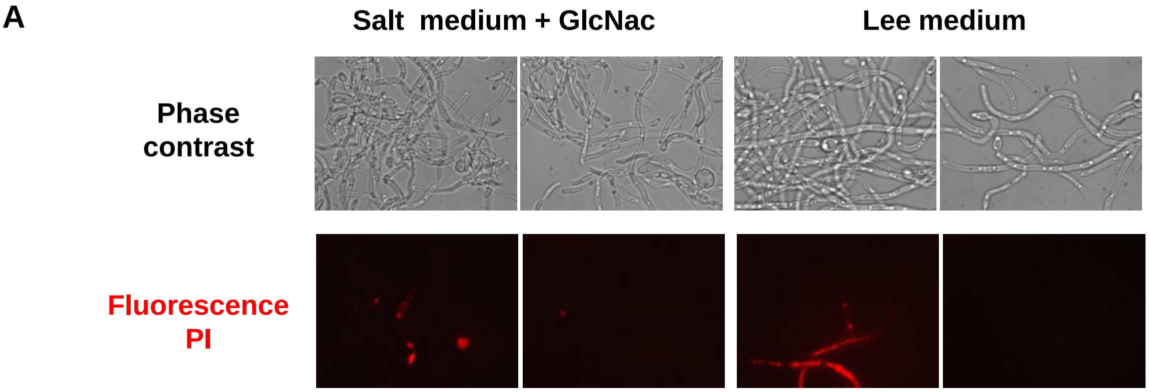

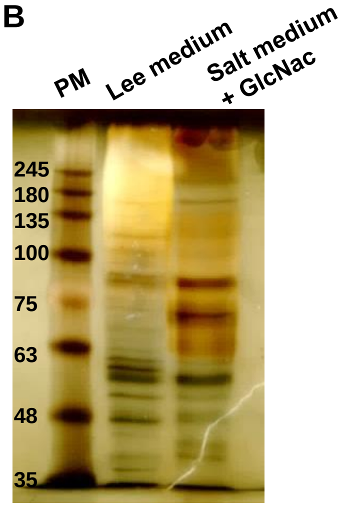

C

Medium	Protein yield from 500 mL-culture (µg)	Unique identified proteins
Salt + GlcNac	17.1	57
Lee (pH 6.7)	16.8	72

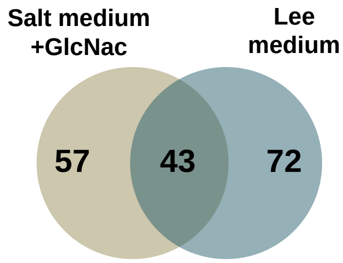

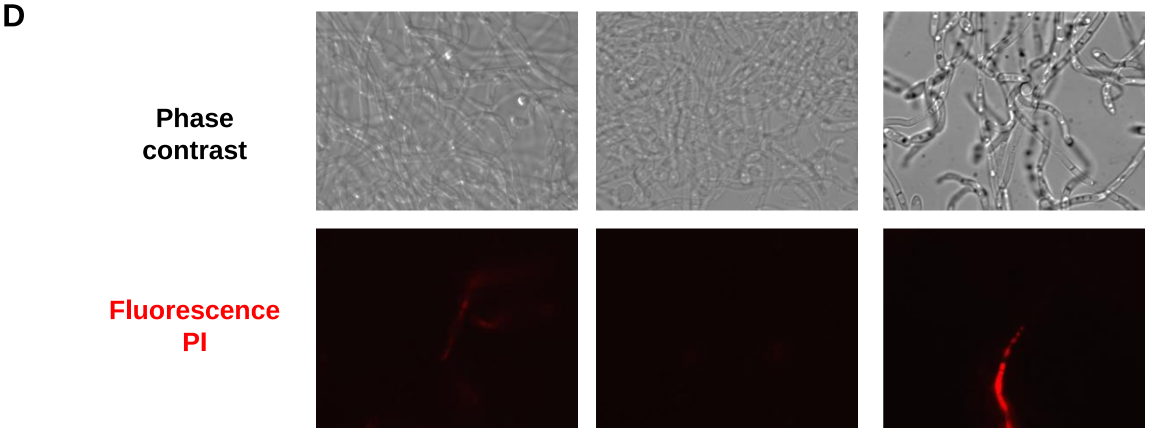

Figure S1. Isolation of the *C. albicans* hyphal secretome in two different growth media. **(A)** Morphology and cell lysis evaluation by propidium iodide (PI) measurement (red fluorescence) in salt medium+GlcNac and Lee medium (pH 6.7). **(B)** Silver-stained SDS-PAGE gel of *C. albicans* hyphal secreted proteins extracted from salt medium+GlcNac and Lee medium (pH 6.7). *PM*, protein marker. **(C)** Comparison of the protein yield and number of proteins identified in salt medium+GlcNac and Lee medium (pH 6.7). Venn diagram shows unique and shared proteins between both media. **(D)** Representative fluorescence images from cell lysis measured by PI staining in *C. albicans* hyphal secretome extraction in Lee medium (pH 6.7) after reducing shaking conditions of cell growth and removing the centrifugation break .

A

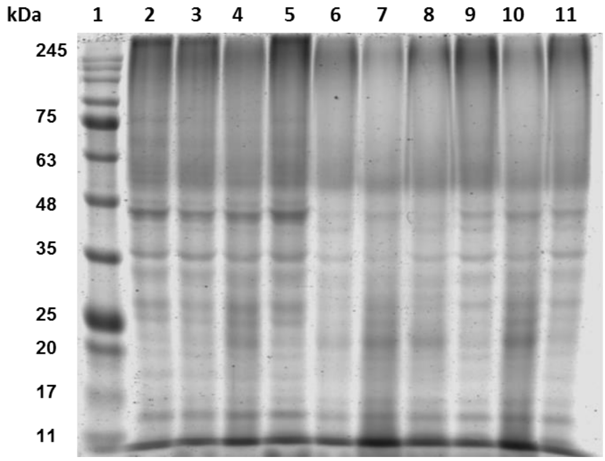

B

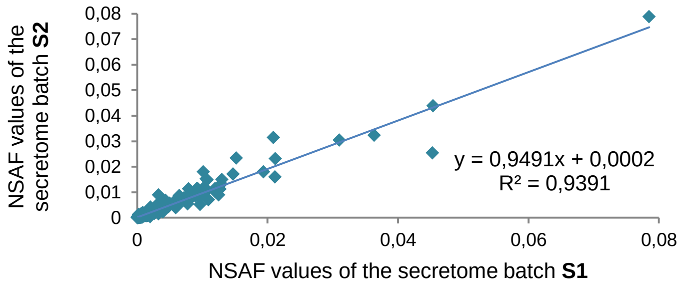

Figure S2. *C. albicans* hyphal secretome samples isolated in Lee medium (pH 6.7). **(A)** Coomassie blue-stained SDS-PAGE gel of the 10 *C. albicans* hyphal secretome samples. An ammount of 10 µg of protein from each extraction was loaded in a 10% SDS-PAGE gel. *Lane 1*, protein marker; *lanes 2-11*, different hyphal secretome samples extracted from 18 h culture of *C. albicans* cells in Lee medium (pH 6.7). **(B)** Correlation of NSAF values of the proteins identified in the two secretome batches (S1 and S2) with at least two peptides by the gel-free LC-MS/MS analysis.

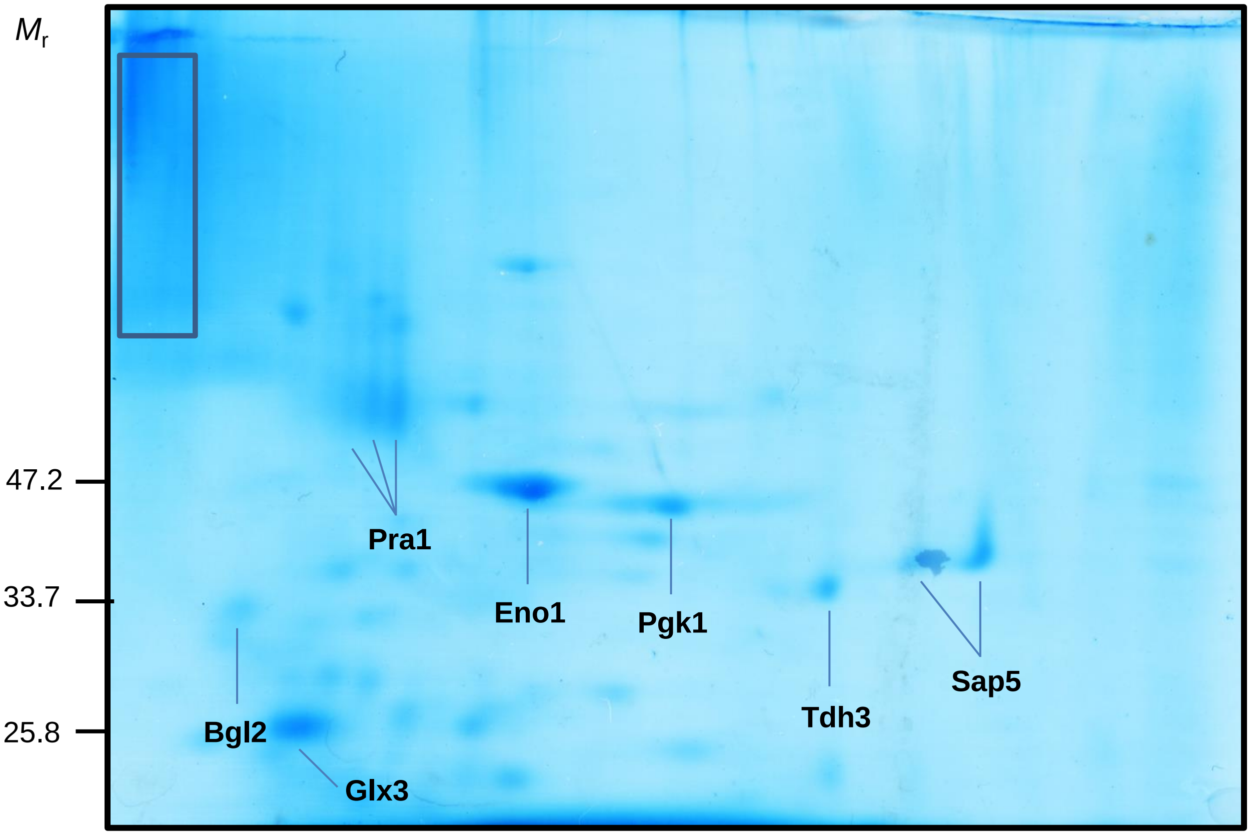

Figure S3. Coomassie blue-stained preparative 2-DE gel of the *C. albicans* hyphal secretome. Preparative gel was used for MALDI-TOF-MS identification. The seven immunoreactive proteins are labeled. The upper-left zone cut for protein identification is depicted with a rectangle.

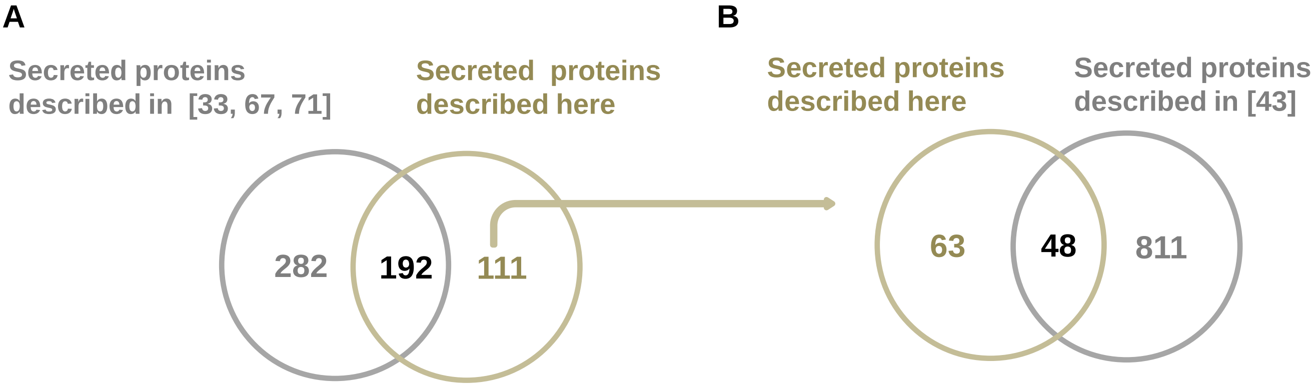

Figure S4. Schematic Venn diagrams comparing *C. albicans* secreted proteins described here and in other studies. **(A)** Comparison between proteins identified in hyphal secretomes here and in previously studies [33, 67, 71]. **(B)** Comparison between proteins identified here and not in the previous studies (111 proteins) with proteins in EVs from hyphal cells [43].
